# Electroconvulsive therapy practice in a general hospital in chile

**DOI:** 10.1192/j.eurpsy.2021.1318

**Published:** 2021-08-13

**Authors:** V. Saitua, N. Schneider, J. Libuy

**Affiliations:** Psiquiatría, Pontificia Universidad Católica de Chile, Santiago, Chile

**Keywords:** psychosis, Electroconvulsive therapy, General Hospital, schizophrénia

## Abstract

**Introduction:**

Electroconvulsive therapy (ECT) is a current, relevant treatment for severe mental illness. In this article we describe our experience on ECT in a public hospital in Chile.

**Objectives:**

Describe a 34 patients’ cohort who received ECT and their outcomes.

**Methods:**

Data was extracted from ECT records between 2018-2020, patients’ evaluations before and after ECT, and case files. Data was then analyzed and described.

**Results:**

ECT was received by 18 males and 16 females. Age ranged from 19 to 73 years (41 average). More than 75% had 12 or more years of education. Patients’ diagnoses and indications for ECT are shown in Figures 1-2

**Figure:**
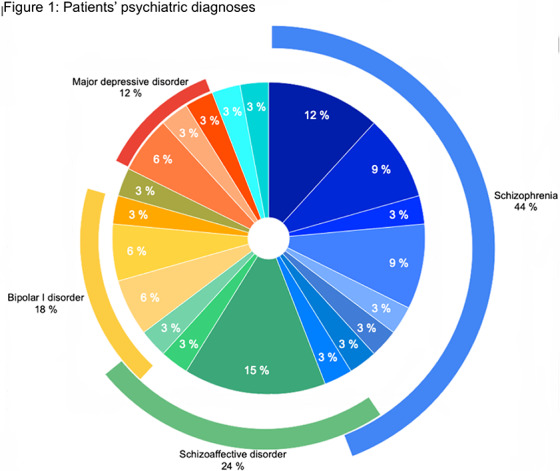

**Figure:**
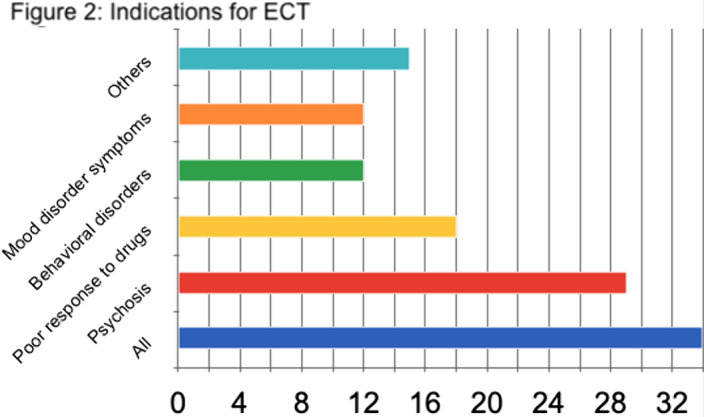

The CGI-SI scale was applied to subjects before treatment, and 85% had a score of 5 or more. On average, patients received 8.7 ECT sessions. Right unilateral electrode placement (RUL) was preferred initially for 94% of patients. Brief pulse width (0.3ms) was most commonly used (76%). Seizure duration averaged in 29 seconds. Adverse reactions presented on 32%, most being mild. One treatment had to be stopped due to confusional symptoms post ECT. After ECT, 91% of patients improved according to CGI-GI scale. 55% were assessed CGI-GI 1 “very much improved”. MoCA scale was also evaluated, showing a 2,1 point gain.

**Conclusions:**

Schizophrenia and psychosis were the most frequent diagnosis and indication for ECT. RUL and brief pulse width were the preferred settings. This cohort suggests that ECT had an impact on markedly ill patients, based on CGI and MoCA scales.

